# Equity in clinical trials for HIV-associated cryptococcal meningitis: A systematic review of global representation and inclusion of patients and researchers

**DOI:** 10.1371/journal.pntd.0009376

**Published:** 2021-05-27

**Authors:** David S. Lawrence, Tshepo Leeme, Mosepele Mosepele, Thomas S. Harrison, Janet Seeley, Joseph N. Jarvis

**Affiliations:** 1 Botswana Harvard AIDS Institute Partnership, Gaborone, Botswana; 2 Department of Clinical Research, Faculty of Infectious and Tropical Diseases, London School of Hygiene and Tropical Medicine, London, United Kingdom; 3 University of Botswana, Gaborone, Botswana; 4 Institute for Infection and Immunity, St George’s University of London, and St George’s University Hospitals NHS Foundation Trust, London, United Kingdom; 5 MRC Centre for Medical Mycology, University of Exeter, Exeter, United Kingdom; 6 MRC/UVRI & LSHTM Uganda Research Unit, Entebbe, Uganda; Yale University School of Medicine, UNITED STATES

## Abstract

**Background:**

It is essential that clinical trial participants are representative of the population under investigation. Using HIV-associated cryptococcal meningitis (CM) as a case study, we conducted a systematic review of clinical trials to determine how inclusive and representative they were both in terms of the affected population and the involvement of local investigators.

**Methods:**

We searched Medline, EMBASE, Cochrane, Africa-Wide, CINAHL Plus, and Web of Science. Data were extracted for 5 domains: study location and design, screening, participants, researchers, and funders. Data were summarised and compared over 3 time periods: pre-antiretroviral therapy (ART) (pre-2000), early ART (2000 to 2009), and established ART (post-2010) using chi-squared and chi-squared for trend. Comparisons were made with global disease burden estimates and a composite reference derived from observational studies.

**Results:**

Thirty-nine trials published between 1990 and 2019 were included. Earlier studies were predominantly conducted in high-income countries (HICs) and recent studies in low- and middle-income countries (LMICs). Most recent studies occurred in high CM incidence countries, but some highly affected countries have not hosted trials. The sex and ART status of participants matched those of the general CM population. Patients with reduced consciousness and those suffering a CM relapse were underrepresented. Authorship had poor representation of women (29% of all authors), particularly as first and final authors. Compared to trials conducted in HICs, trials conducted in LMICs were more likely to include female authors (32% versus 20% *p* = 0.014) but less likely to have authors resident in (75% versus 100%, *p* < 0.001) or nationals (61% versus 93%, *p* < 0.001) of the trial location.

**Conclusions:**

There has been a marked shift in CM trials over the course of the HIV epidemic. Trials are primarily performed in locations and populations that reflect the burden of disease, but severe and relapse cases are underrepresented. Most CM trials now take place in LMICs, but the research is primarily funded and led by individuals and institutions from HICs.

## Author summary

It is essential that clinical trial participants are representative of the population under investigation. Similarly, research must meaningfully include researchers who are from and/or based in the location where the study is being conducted, both to ensure that the research matches the local need but also to promote equity in research. Using clinical trials in HIV-associated cryptococcal meningitis as a case study, we conducted a systematic review to determine how inclusive and representative trials have been across the course of the HIV epidemic. We identified 39 studies. There was a geographical shift with trials moving from the USA to Africa and Asia over time. We found that recent trials were conducted in areas heavily affected by cryptococcal meningitis, but we did identify geographical areas and patient groups that have been underrepresented. We also found inequality within authorship that was skewed towards male researchers from high-income countries. These findings outline areas for our discipline to focus on. We can also use this study as a benchmark from which to monitor our progress over time. This is a broad methodology that could be adopted and adapted by other research groups.

## Introduction

Global health research is a rapidly expanding and evolving field, and global health practitioners are increasingly reflecting on the ethos and equity of research [1–3]. Concerns have been raised that clinical trials are disproportionately conducted in a limited number of countries [4–7] and that individual researchers and institutions from high-income countries (HICs) disproportionately benefit from global health research [8], leading to calls to examine and potentially reform how global health research is conceptualised and conducted.

The emergence of the HIV epidemic was a catalyst for huge investment in global health, both in terms of research and service provision [9]. This expansion in investment led to the creation of a large number of transnational research partnerships (TRPs), whereby institutions in HICs partnered with those in low- and middle-income countries (LMICs) to collaborate on research studies [10]. As a consequence, an increasing number of research publications in the field of HIV are published each year, and the number of countries contributing to HIV research continues to grow [11]. This research has contributed to dramatic reductions in the number of new infections and deaths due to HIV over the last 30 years. Despite this, an estimated 1,700,000 people were newly infected with HIV, and 690,000 AIDS-related deaths occurred in 2019 [12], with sub-Saharan Africa at the centre of the epidemic. There remains a clear need for further research in prevention, treatment, and implementation. Ongoing research needs to be appropriate to the research setting, address high-priority research questions [13], and include representative patient populations in order to generate applicable and generalisable findings [14].

In addition to ensuring that research participants are representative of the general population suffering from a disease, it is equally important to ensure that the researchers and institutions involved are representative of where the disease burden lies. At present, the majority of HIV funding comes from HICs [9], and therefore, the resources (both economic and human) flow from HICs to LMICs. Individuals and institutions from HICs lead the research, and researchers from LMICs are often found in the middle of author lists or excluded altogether from publications arising from African health research [15]. Inclusive research teams are essential to shape priorities and develop studies based on an in-depth understanding of the local context and inclusive representation will promote fairness, strengthen capacity, and ensure the future sustainability of research.

Cryptococcal meningitis (CM) remains a significant contributor to AIDS-related mortality in LMICs despite expanding rollout of effective antiretroviral therapy (ART) [16,17]. Annual global deaths from CM are estimated at 181,000, and CM is responsible for 15% of all AIDS-related deaths [18]. Over the past 30 years, clinical trials have defined treatment strategies that have led to a dramatic reduction in 10-week mortality rates from almost 100% to approximately 30% to 40% [19–21], but further clinical trials remain essential to further improve mortality outcomes and develop treatments appropriate for LMICs. Using CM as a case study, we performed a systematic review to examine how representative and inclusive CM clinical trials have been over the course of the HIV epidemic. Our aim was to describe the location of CM trials and the characteristics of those enrolled in order to perform a comparison with the current epidemiology. We also aimed to describe the gender, location, and nationality of researchers involved in CM trials.

## Methods

We included any trial in which individuals with HIV-associated CM were randomly assigned to 1 of at least 2 intervention arms. The intervention could be any treatment for their condition, and there was no restriction on the nature of the comparator arm. Our focus was on the characteristics of individuals who were recruited into the trials and the researchers conducting the studies, and not on trial outcomes.

### Search method and data collection

We searched for studies published up to 4 March 2020 using Medline, EMBASE, Cochrane Library, Africa-Wide, CINAHL Plus, and Web of Science. We also searched ClinicalTrials.gov for completed and published trials. Our search strategy combined terms related to HIV-associated CM and clinical trials (see Table 1A). No restrictions were placed on language. We excluded studies related to CM that was not associated with HIV or where data from HIV-infected individuals could not be extracted, observational studies, healthy volunteer studies recruiting participants with previously treated CM, studies without comparator arms, manuscripts where data were presented elsewhere in a primary manuscript, and nonoriginal research articles such as editorials. The search strategy and protocol were developed by the authors prior to commencing the search and were registered with PROSPERO (CRD42020171845).

**Table 1:** (A) The search strategy. (B) A summary of the variables extracted from included papers.

**A) Search strategy**			
#1	Search (Meningitis, Cryptococcal[Mesh] OR cryptococcal meningitis)		
#2	Search (trial[mesh] OR Clinical Trial OR Clinical Trial, Phase I OR Clinical Trial, Phase II OR Clinical Trial, Phase III OR Clinical Trial, Phase IV OR Randomized Controlled Trial)		
#3	Search (Prospective Studies[Mesh] or prospective)		
	Searching #1 and #2 and #3 up to and including 04 March 2020		
**B) Variables extracted from included papers**			
**Study**		• Year of publication • Period of study • Location of study • Type of healthcare facility • Study design • Intervention(s)	• Control • Inclusion criteria • Exclusion criteria • Primary outcome • Secondary outcome(s)
**Screening and Randomisation**		• Number screened • Number screen failures • Reasons for each	• Withdrawals • Loss to follow-up
**Participants**		• Number of participants • Gender • Antiretroviral status	• Baseline Glasgow Coma Scale • First episode or relapse
**Researchers**		• Number of authors • Gender • Country of origin	• Country of residence during research period
**Funders**		• Name of funders • Category of funder	• Location of funder • Funding amount

All papers were entered into Covidence [22]. Duplicates were removed and then titles and abstracts were independently screened against the eligibility criteria by DSL and TL. Noneligible studies were removed, and the full texts of potentially eligible titles were assessed for inclusion. JNJ and MM adjudicated in the case of any conflict regarding study inclusion. The reference lists of included studies were searched to identify any additional eligible studies.

### Data extraction

We extracted the relevant variables from each included paper (Table 1B) in 5 key domains: Study location and design, screening, participants, researchers, and funders. If necessary, the authors of an article were contacted for information that may not have been presented in the final publication. Researcher data were augmented by online searches of institutional webpages and profiles on sites such as LinkedIn and Research Gate. If gender data could not be confidently elicited, then the gender of authors was determined using a website called Genderize.io that predicts the gender of a person given their name. Either DSL or TL performed the data extraction and then the other verified the data. Any discrepancies were discussed and resolved.

### Data synthesis and analysis

Data were summarised using descriptive statistical analysis. To describe the geography of where participants were recruited, the locations of trial sites were analysed individually and also grouped into World Bank Regions. To demonstrate trends over time, comparison was made over 3 different periods: pre-2000, 2000 to 2009, and post-2010 to broadly demonstrate the pre-widespread ART era, early ART era, and established ART era, respectively. The end date of recruitment was used to determine within which of these time periods the study would be categorised. In papers where the specific months of recruitment were not stated, the year of publication was used. Where data could not be extracted for individual sites within multicountry trials, these numbers were averaged. We compared the characteristics of trial participants (gender, relapse rate, ART status, and baseline Glasgow Coma Scale (GCS)) to a composite reference of recently published observational and surveillance data from routine care settings[16,23–26]. ART experienced was defined as being on ART at the time of randomisation, including individuals who were on zidovudine monotherapy prior to the availability of combination ART. Chi-squared testing for trend was performed to describe trends in the demographics of trial participants and researchers over time. With regard to the gender, countries where researchers were born and where they resided during the trial, each study was categorised as either taking place in HICs or LMICs, and chi-squared calculations allowed comparison between these 2 groups. Statistical analysis was conducted in Stata/SE 15.0.

## Results

**Fig 1:**
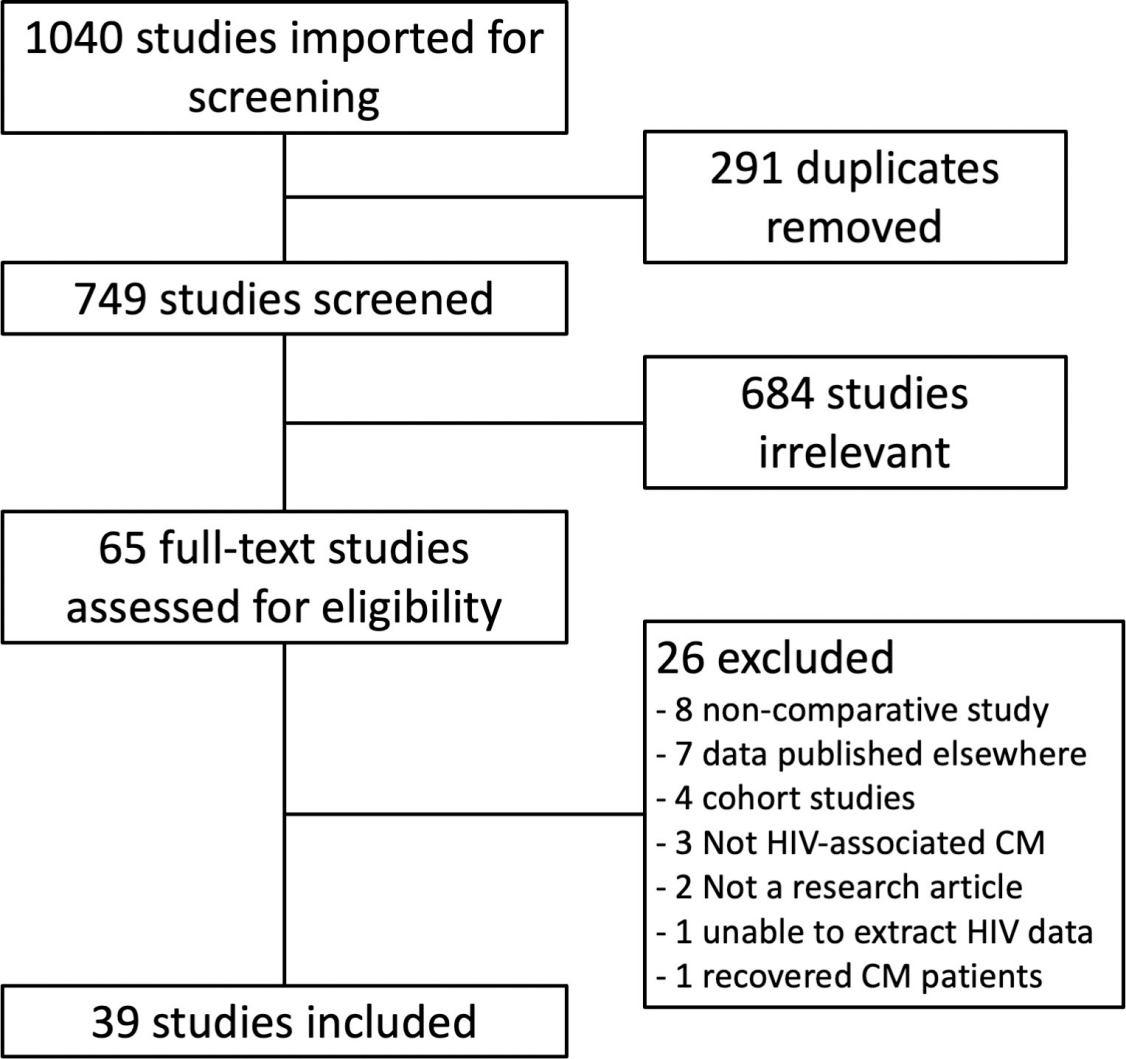
PRISMA diagram.

The initial database search yielded 1,040 studies (Fig 1), of which 291 were duplicates. A total of 749 titles and abstracts were reviewed, with 65 selected for full-text review. Of these, 26 were excluded. No additional studies were identified after reviewing the reference lists of included studies. A total of 39 studies were included in the final data analysis (Table 2).

**Table 2:** A summary of the 39 included studies.

Study	Title
Larsen 1990 [27]	Fluconazole compared with amphotericin B plus flucytosine for cryptococcal meningitis in AIDS. A randomized trial.
Bozzette 1991 [28]	A placebo-controlled trial of maintenance therapy with fluconazole after treatment of cryptococcal meningitis in the acquired immunodeficiency syndrome.
deGans 1992 [29]	Itraconazole compared with amphotericin B plus flucytosine in AIDS patients with cryptococcal meningitis.
Saag 1992 [30]	Comparison of amphotericin B with fluconazole in the treatment of acute AIDS-associated cryptococcal meningitis.
Powderly 1992 [31]	A controlled trial of fluconazole or amphotericin B to prevent relapse of cryptococcal meningitis in patients with the acquired immunodeficiency syndrome.
Sharkey 1996 [32]	Amphotericin B lipid complex compared with amphotericin B in the treatment of cryptococcal meningitis in patients with AIDS.
Joly 1996 [33]	Randomized comparison of amphotericin B deoxycholate dissolved in dextrose or Intralipid for the treatment of AIDS-associated cryptococcal meningitis.
Leenders 1997 [34]	Liposomal amphotericin B (AmBisome) compared with amphotericin B both followed by oral fluconazole in the treatment of AIDS-associated cryptococcal meningitis.
Chotmongkol 1997 [35]	Comparison of amphotericin B, flucytosine and itraconazole with amphotericin B and flucytosine in the treatment of cryptococcal meningitis in AIDS.
Van der Horst 1997 [36]	Treatment of cryptococcal meningitis associated with the acquired immunodeficiency syndrome.
Mayanja-Kizza 1998 [37]	Combination therapy with fluconazole and flucytosine for cryptococcal meningitis in Ugandan patients with AIDS.
Saag 1999 [38]	A comparison of itraconazole versus fluconazole as maintenance therapy for AIDS-associated cryptococcal meningitis.
Newton 2002 [39]	A randomized, double-blind, placebo-controlled trial of acetazolamide for the treatment of elevated intracranial pressure in cryptococcal meningitis.
Vibhagool 2003 [40]	Discontinuation of secondary prophylaxis for cryptococcal meningitis in human immunodeficiency virus-infected patients treated with highly active antiretroviral therapy: a prospective, multicenter, randomized study.
Mootsikapun 2003 [41]	The efficacy of fluconazole 600 mg/day versus itraconazole 600 mg/day as consolidation therapy of cryptococcal meningitis in AIDS patients.
Pappas 2004 [42]	Recombinant interferon- gamma 1b as adjunctive therapy for AIDS-related acute cryptococcal meningitis.
Brouwer 2004 [43]	Combination antifungal therapies for HIV-associated cryptococcal meningitis: a randomised trial.
Chotmongkol 2005 [44]	Initial treatment of cryptococcal meningitis in AIDS.
Tansuphaswadikul 2006 [45]	Comparison of one week with two week regimens of amphotericin B both followed by fluconazole in the treatment of cryptococcal meningitis among AIDS patients.
Techapornroong 2007 [46]	Alternate-day versus once-daily administration of amphotericin B in the treatment of cryptococcal meningitis: a randomized controlled trial.
Milefchik 2008 [47]	Fluconazole alone or combined with flucytosine for the treatment of AIDS-associated cryptococcal meningitis.
Bicanic 2008 [48]	High-dose amphotericin B with flucytosine for the treatment of cryptococcal meningitis in HIV-infected patients: a randomized trial.
Pappas 2009 [49]	A phase II randomized trial of amphotericin B alone or combined with fluconazole in the treatment of HIV-associated cryptococcal meningitis.
Nussbaum 2010 [50]	Combination flucytosine and high-dose fluconazole compared with fluconazole monotherapy for the treatment of cryptococcal meningitis: a randomized trial in Malawi.
Makadzange 2010 [51]	Early versus delayed initiation of antiretroviral therapy for concurrent HIV infection and cryptococcal meningitis in sub-Saharan Africa.
Jadhav 2010 [52]	Liposomal amphotericin B (FungisomeTM) for the treatment of cryptococcal meningitis in HIV/AIDS patients in India: A multicentric, randomized controlled trial
Hamill 2010 [53]	Comparison of 2 doses of liposomal amphotericin B and conventional amphotericin B deoxycholate for treatment of AIDS-associated acute cryptococcal meningitis: a randomized, double-blind clinical trial of efficacy and safety.
Loyse 2012 [54]	Comparison of the early fungicidal activity of high-dose fluconazole, voriconazole, and flucytosine as second-line drugs given in combination with amphotericin B for the treatment of HIV-associated cryptococcal meningitis.
Jarvis 2012 [55]	Adjunctive interferon-gamma immunotherapy for the treatment of HIV-associated cryptococcal meningitis: a randomized controlled trial.
Jackson 2012 [56]	A phase II randomized controlled trial adding oral flucytosine to high-dose fluconazole, with short-course amphotericin B, for cryptococcal meningitis.
Day 2013 [57]	Combination antifungal therapy for cryptococcal meningitis.
Bisson 2013 [58]	Early versus delayed antiretroviral therapy and cerebrospinal fluid fungal clearance in adults with HIV and cryptococcal meningitis.
Boulware 2014 [59]	Timing of antiretroviral therapy after diagnosis of cryptococcal meningitis.
Vaidhya 2015 [60]	Combination versus monotherapy for the treatment of HIV associated cryptococcal meningitis
Beardsley 2016 [21]	Adjunctive Dexamethasone in HIV-Associated Cryptococcal Meningitis.
Villanueva-Lozano 2018 [61]	Clinical evaluation of the antifungal effect of sertraline in the treatment of cryptococcal meningitis in HIV patients: a single Mexican centre experience.
Molloy 2018 [19]	Antifungal Combinations for Treatment of Cryptococcal Meningitis in Africa.
Rhein 2019 [62]	Adjunctive sertraline for HIV-associated cryptococcal meningitis: a randomised, placebo-controlled, double-blind phase 3 trial
Jarvis 2019 [63]	Short-course High-dose Liposomal Amphotericin B for Human Immunodeficiency Virus-associated Cryptococcal Meningitis: A Phase 2 Randomized Controlled Trial

### Study design and location

**Fig 2:**
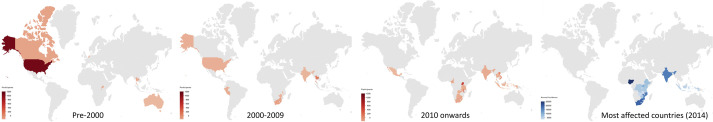
Number of participants recruited into HIV-associated cryptococcal meningitis cryptococcal trials by country and broken down into time periods: (A) pre-2000, (B) 2000–2009, (C) 2010 onwards, and D) data from the global disease burden estimates identifying the 12 countries globally with the largest number of annual cases in 2014 [18]. Created using a base map available at www.displayr.com/create-a-geographic-map/.

We identified 39 trials that recruited a total of 5,056 participants between 1985 and 2017 and were published between 1990 and 2019 (Table 3). Fig 2 highlights the location and number of participants that were recruited into clinical trials during the different time periods and presents this in comparison with data from the comprehensive 2014 global burden of disease estimates highlighting the 12 countries with the largest annual number of incident cases [18]. During the 30 years covered by this review, trials have moved from predominantly being conducted in the United States of America to sub-Saharan Africa where 75% of all CM trial participants were recruited since 2010. Only 43 patients from Europe and Central Asia have been recruited into a CM trial. The majority of trials (*n =* 29 (74%)) focused on induction therapy for CM. Primary outcomes were initially centred around clinical and microbiological markers, but in recent years, there has been an increasing trend towards mortality being the primary outcome. The majority (*n* = 38 (97%)) of trials only included HIV–infected patients and, where data were available, 20/24 (83%) trials only permitted the inclusion of patients suffering from a first episode of CM and 9/20 (45%) permitted only ART-naïve patients to be included. No studies specifically stated that pregnant or lactating women could be included but 30 (77%) and 23 (59%) studies, respectively, explicitly excluded these populations.

**Table 3:** Characteristics of HIV-associated cryptococcal meningitis trials within different time periods and overall. Where data were not available, the number of trials with available data is presented as a denominator.

		PRE-2000	2000–2009	2010 ONWARDS	OVERALL N(%)	
**TRIAL DESIGN**						
**Number of trials**		14	14	11	39	
**Focus of trial**	**Induction**	10 (71%)	10 (71%)	9 (82%)	29 (74%)	
	**Maintenance**	3 (21%)	2 (14%)	0	5 (13%)	
	**ART timing**	0	1 (7%)	2 (18%)	3 (8%)	
	**Other**	1 (7%)	1 (7%)	0	2 (5%)	
**Primary outcome**	**Mortality**	0	1 (7%)	6 (55%)	7 (18%)	
	**Microbiological**	4 (29%)	7 (50%)	5 (45%)	16 (41%)	
	**Clinical**	3 (21%)	6 (43%)	0	9 (23%)	
	**Combined**	7 (50%)	0	0	7 (18%)	
**Inclusion**	**HIV infected only**	14 (100%)	13 (93%)	11 (100%)	38 (97%)	
	**First episode only**	4/7 (57%)	9/10 (90%)	7/7 (100%)	20/24 (83%)	
	**ART naïve only**	0/5	5/6 (83%)	4/9 (44%)	9/20 (45%)	
**SCREENING AND RANDOMISATION**						
**Number of studies with data**		1	7	10	18	
**Number screened**		42	965	4,004	5,011	
**Number screened out**		16	589	1,643	2,248	
**% screen failures**		38%	61%	41%	45%	
**Screen failures**	**Declined**	6 (14%)	66 (7%)	292 (7%)	364 (7%)	
	**Pregnant**	0	2 (0.2%)	10 (0.2%)	12 (0.2%)	
	**Lactating**	0	0	5 (0.1%)	5 (0.1%)	
**Number randomised**		1,814	797	2,455	5,066	
**Withdrawal**	**Number of studies with data**	13	14	11	38	
	**Number randomised**	1,759	797	2,455	5,011	
	**Late exclusion**	132 (8%)	26 (3%)	50 (2%)	208 (4%)	
	**Withdrawal of consent**	24 (1%)	3 (0.4%)	8 (0.3%)	35 (1%)	
	**Loss to follow-up**	58 (3%)	48 (6%)	16 (0.7%)	122 (2%)	
**PARTICIPANTS**						***P* value**
**Gender**	**Number of studies with data**	13	14	11	38	
	**Number male**	1,454	480	1,494	3,427	
	**Number female**	183	285	901	1,369	
	**% female**	11%	37%	38%	29%	*p* < 0.0001*
**ART status**	**Number of studies with data**	7	9	9	25	
	**Number ART naïve**	906	594	1,196	2,696	
	**Number ART experienced**	288	11	889	1,188	
	**% ART experienced**	24%	2%	43%	31%	*p* < 0.0001*
**Episode**	**Number of studies with data**	6	9	7	22	
	**Number first episode**	997	557	1,822	3,376	
	**Number relapse**	28	0	0	28	
	**% relapse**	3%	0%	0%	1%	*p* < 0.0001*
**Baseline GCS**	**Number of studies with data**	8	9	9	26	
	**Number GCS 15**	765	410	1,654	2,829	
	**Number GCS <15**	131	58	697	886	
	**% number GCS <15**	15%	12%	30%	24%	*p* < 0.0001*

ART, antiretroviral therapy; GCS, Glasgow Coma Scale.

*Statistically significant.

### Screening and randomisation

Screening data were not available for all studies but were reported more thoroughly in recently published papers. Out of 18 papers where data were available, a total of 5,011 potential participants were screened, and 2,763 (55%) were randomised. Seven percent of individuals that were approached declined consent, and this was consistent across time periods. There were 12 documented instances of a patient not being included due to pregnancy and 5 due to lactation. The majority of studies (*n =* 38 (97%)) reported data on those who were withdrawn due to meeting an early withdrawal criteria (*n =* 208/5,011 (4%)), withdrew their consent (*n =* 35/5,011 (1%)), or were lost to follow-up (*n =* 122/5,011 (2%)).

### Participants

**Fig 3:**
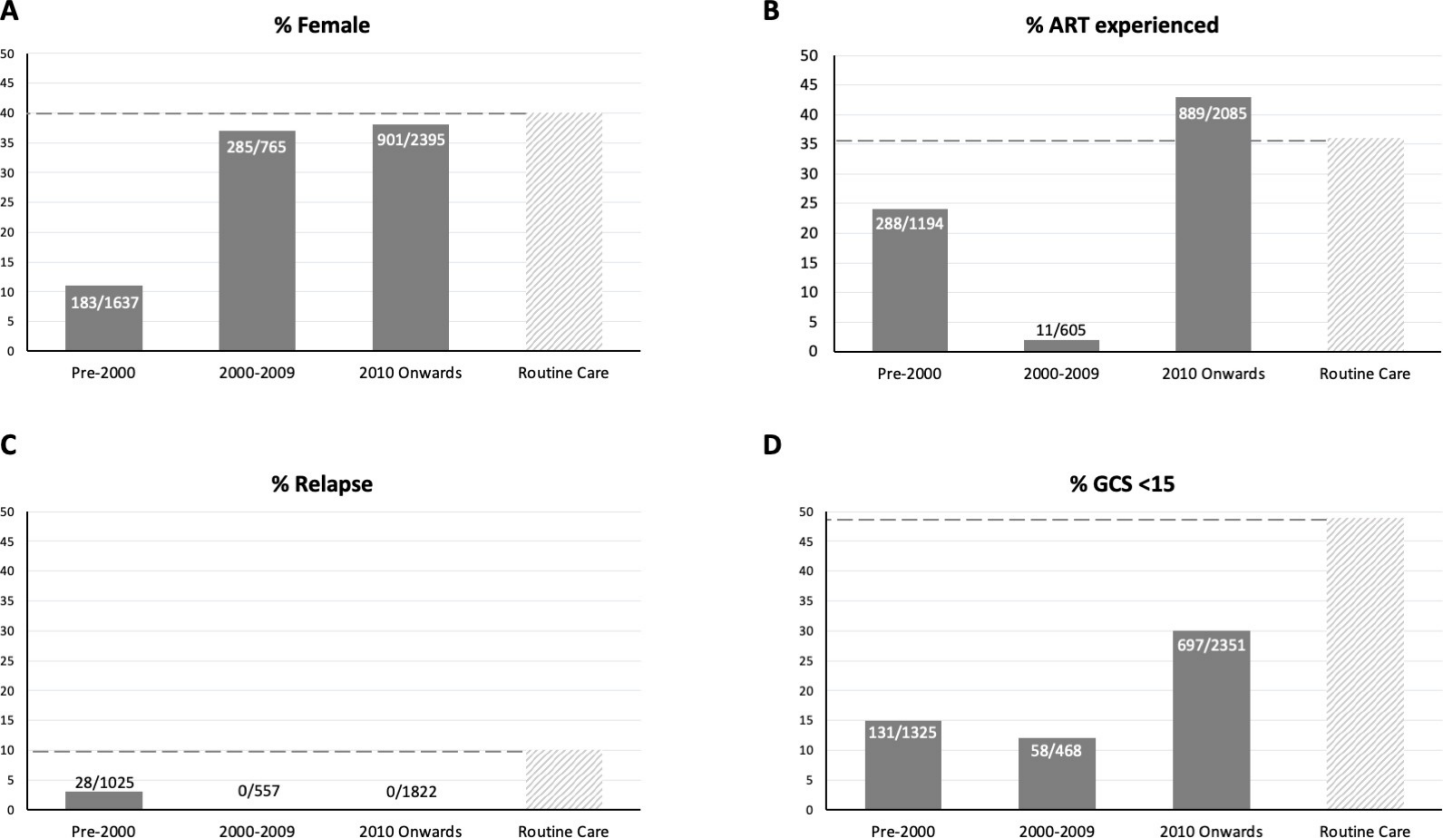
The characteristics of HIV-associated cryptococcal meningitis trial participants across 3 different time periods (pre-2000, 2000–2009, and 2010 onwards) broken down by (A) Sex, (B) ART experience, (C) Relapse, and (D) Baseline Glasgow Coma Scale (15) score, all compared with a composite reference from recently published observational data.

Data from 38/39 included studies demonstrated that 1,369/4,796 (29%) of participants in CM trials were female (Fig 3). There was a significant increase over time in the proportion of female participants (*p* < 0.0001). There was no report of any pregnant or lactating women being included in any clinical trial. A total of 1,188/3,884 (31%) participants were ART experienced upon enrolment into the trial. The proportion of ART experienced participants fluctuated from 24% prior to 2000 to 2% between 2000 and 2009 and 43% after 2010 demonstrating a general increase (*p* < 0.0001). A total of 28/3,404 (0.8%) participants were presenting with a relapse of CM, and these were all recruited prior to 2000. Twenty-four percent (886/3,715) of participants were recruited with a GCS <15, indicating impaired decision-making capacity, and there was a trend for the proportion with reduced GCS to increase over time (*p* < 0.0001).

### Researchers

The median number of researchers named as authors on the primary manuscript was 11 (range 2 to 37), and 7 papers also had additional contributors listed within an appendix (Table 4). Of all named researchers, 29% were women. Overall, female researchers were underrepresented as first (26%), second (29%), third (19%), and final author (13%). No significant change was observed over time. Regarding whether named authors were resident in one of the research locations during the period of the study, this was case for the majority of first (95%), second (89%), and final (74%) authors. A total of 82% of all named authors were resident in one of the research locations, and there was a trend for this proportion to reduce over time (*p* < 0.0001). Finally, in terms of whether authors were nationals of countries where participants were being recruited, this was the case for a smaller majority of first (59%), second (84%), and final (67%) of authors. A total of 70% of named authors were nationals of research sites, and there was a strong downward trend observed over time (*p* < 0.0001). When comparing these same 3 domains by comparing studies that were conducted in HICs and LMICs, there were significantly more female authors on studies conducted in LMICs (*p* = 0.014). There were also significantly fewer named authors who were resident or nationals of research sites within trials conducted in LMICs (*p* = < 0.0001 and *p* = < 0.0001, respectively).

**Table 4:** Researcher data summarising the number, gender, country of residence, and nationality of named authors on the primary manuscript of HIV-associated cryptococcal meningitis clinical trials.

		PRE-2000	2000–2009		2010 ONWARDS		OVERALL		
Number of papers		14	14		11		39		
Median number of authors (range)		12 (3–17)	8 (2–14)		17 (4–37)		11 (2–37)		
**POSITION IN LIST OF AUTHORS**									***P* value**
First author	number female (%)	3 (21%)	6 (43%)		1 (9%)		10 (26%)		*p* = 0.5712
	Number resident of research location (%)	13 (93%)	14 (100%)		10 (91%)		37 (95%)		*p* = 0.0885
	Number national of research location (%)	12 (86%)	9 (64%)		2 (18%)		23 (59%)		*p* = 0.0008*
Second author	number female (%)	2 (14%)	5/13 (38%)		4 (36%)		11/38 (29%)		*p* = 0.2037
	Number resident of research location (%)	12 (86%)	12/13 (92%)		10 (91%)		34/38 (89%)		*p* = 0.6541
	Number national of research location (%)	12 (86%)	12/13 (92%)		8 (73%)		32/38 (84%)		*p* = 0.4167
Final author	number female (%)	2 (14%)	3 (21%)		0		5 (13%)		*p* = 0.3316
	Number resident of research location (%)	12 (86%)	10 (71%)		7 (64%)		29 (74%)		*p* = 0.2026
	Number national of research location (%)	12 (86%)	9 (64%)		5 (45%)		26 (67%)		*p* = 0.0331*
**OF ALL NAMED AUTHORS**									
Number of named authors		147	116		193		456		
Gender balance	number female (%)	31 (21%)	44 (38%)		58 (30%)		133 (29%)		*p* = 0.1027
Residence	Number resident of research location (%)	132 (90%)	99 (85%)		141 (73%)		372 (82%)		*p* < 0.0001*
Nationality	Number national of research location (%)	124 (84%)	88 (76%)		105 (54%)		317 (70%)		*p* < 0.0001*
**OF ALL NAMED AUTHORISED CATEGORISED BY INCOME STATUS OF RESEARCH LOCATION**									
		**HIC**		**LMIC**		**OVERALL**		***P* value**	
Number of papers		11		28		39			
Number of named authors		122		334		456			
Gender balance	number female (%)	25 (20%)		108 (32%)		133 (29%)		*p* = 0.014*	
Residence	Number resident of research location (%)	122 (100%)		250 (75%)		372 (82%)		*p* < 0.0001*	
Nationality	Number national of research location (%)	114 (93%)		203 (61%)		317 (70%)		*p* < 0.0001*	

HICs, high-income countries; LMICs, low- to middle-income countries.

*Statistically significant.

### Funding

There were significant missing data related to the funding of trials, particularly the funding amount. Of the 33 trials where data were available, 19 (58%) were funded by a single funder, and 14 (42%) had multiple funders. Thirty-six percent of trials had funding from industry, 67% from government bodies, and 42% from nongovernmental bodies. Twenty-nine (88%) trials were entirely funded by institutions based in HICs, and 4 (12%) were entirely funded by institutions based in LMICs.

## Discussion

Our systematic review findings show that HIV-associated CM trials are generally conducted in locations reflecting the global burden of disease. There has been a marked shift from the USA to sub-Saharan Africa over the last 2 decades. CM trials are broadly representative of the patient population, but there is an underrepresentation of very sick patients with a low baseline GCS and those suffering with a relapse of CM. With the change in location from HICs to LMICs, there has been a significant trend for authors to be nonnationals of the country where research is performed, particularly in first and senior author positions. Female authors are generally underrepresented and, again, this is most marked in first and particularly senior author positions.

CM remains a major public health problem, and it is reassuring that the number of trials being performed has remained steady throughout the HIV epidemic. The bulk of CM disease has likely always been in sub-Saharan Africa, and roughly 75% of all CM deaths currently occur in sub-Saharan Africa. As the number of cases of CM reduced in North America and Western Europe, there was a shift in focus to LMICs with a clear desire to identify simpler, less toxic, and more effective treatment regimens for CM. It is therefore appropriate that sub-Saharan Africa is now the epicentre of CM trials, recruiting 76% of all trial participants in the last decade. This is followed by the Asia and Pacific region where 22% of all CM deaths occur and 21% of participants were recruited. This demonstrates that the regional distribution of CM trials is now well matched to the epidemiology and likely mirrors a general increase in funding for global health [64], including HIV research in LMICs [65]. The bulk of research has taken place in a small number of countries (particularly Malawi, South Africa, and Uganda). By studying Fig 2, which compares the number of participants recruited in different countries with data from the global disease burden estimates identifying the 12 countries globally with the largest number of annual cases in 2014, one can see areas with a high burden of disease that have not been involved in clinical trials such as Nigeria, Kenya, and Mozambique. Other countries, such as India for example, have a lot of CM cases but have only recruited a small number of participants into clinical trials. This uneven geographical spread is not unique to CM trials and is reflected in the global distribution of clinical trials more broadly. There are multiple, overlapping reasons for this which include the levels of internal and external funding made available to research and development in a specific country [66]; access to partnerships with other research institutions; experience with clinical research (including having hosted previous CM trials); and the efficiency of the regulatory approval process in country [67].

When considering the participants in CM trials, the proportion who are female, ART experienced, and suffering with severe disease has increased over time. The majority of earlier trials were conducted in HICs where the epidemic particularly affected men who have sex with men, and over time as trials were increasingly conducted in sub-Saharan Africa and the Asia and Pacific region, there has been an increase in the proportion of female participants: 38% of all trial participants recruited since 2010 were female. Men are more likely to be diagnosed with advanced HIV disease, either due to delayed testing or nonadherence to ART and therefore more likely to develop CM. In routine care conditions, the proportion of CM patients who are female is roughly 40% [16,23,24,68], so although there is no gender parity, the most recent CM trials mirror the general patient population.

CM almost entirely occurs in individuals with very advanced HIV disease (CD4 <100 cells/mm^3^), and conception is quite rare in this population [69]. Pregnant and breastfeeding women are however most often excluded from CM trials due to a number of reasons. Often there is reluctance from ethics committees to include pregnant women in research studies, and sponsoring institutions may not be willing to take on the risk of litigation in the event of a poor outcome [70]. In addition, there are scientific concerns about antifungal toxicity, particularly with regard to fluconazole as there is weak evidence to suggest it is teratogenic at the high doses given for CM [71,72]. One caveat to this is whereby trials include lactating women who voluntarily stop breastfeeding to participate in the trial and are supplied with formula milk by the research team. In routine care settings, pregnant and lactating women with CM often receive treatment with fluconazole, particularly where this is the only treatment available. The inclusion of pregnant and lactating women in clinical trials is a broad and urgent issue [2,73,74], and there is reassuring evidence to suggest that this deficit is slowly being addressed in HIV-related clinical trials at least. Our research has identified that, out of 5,011 patients who were screened, only 12 were excluded due to pregnancy and 5 due to lactation, so this is not a large population that is being excluded. That does however not negate the need to build an evidence base to guide the management of pregnant and lactating women suffering from this potentially fatal infection. The most comprehensive collection of clinical data on CM in pregnancy is a case series from Uganda [75]. Teams across the globe should strongly consider collecting and combining observational data sets to build a deeper understanding of the safety profile of different treatment regimens and to collate maternal and neonatal outcomes. In addition, pregnant and lactating women should be considered for inclusion in future trials.

Despite allowing treatment-experienced patients (those predominantly on zidovudine monotherapy) to be recruited in the earlier trials in the USA, concerns about ART status and the potential for that to be a confounding factor led to most trials in the 2000s only including ART-naïve patients. These concerns proved to be unfounded, and there has been no difference in outcome observed between these groups [19]. Over the last decade, the number of CM patients who were ART experienced has steadily increased to the current levels seen today of roughly 36% [23,24,26], and clinical trial participation has matched this.

Importantly, almost all trials have excluded patients suffering from a relapse of CM. Only 28 individuals in total were recruited into trials, and these were all before 2000. The rationale for this is that patients may have acquired antifungal resistance and that the induction regimens may not be effective. The 2 largest CM trials conducted in recent years excluded 8% to 9% of all patients screened due to relapse [19,62], and recent published data from routine care settings have shown relapse to occur in roughly 10% of cases [16,23,25,26]. Relapse is therefore relatively common and drug resistance testing is not widely available so this population should be considered for future clinical trials, either among those suffering a first episode or within independent studies.

The methodology adopted in this study can also assist with the planning of future clinical trials and the assumptions that can be made about participant attrition when calculating a sample size. We have learned that it is rare for participants to withdraw their consent (1%) or be lost to follow-up (2%). This likely reflects the fact that CM is a life-threatening condition and clinical trials often provide promising therapies or, particularly in LMICs, a standard of care that is superior to the routine care available. There is extensive literature to demonstrate that individuals with CM who enrol in a clinical trial have a better outcome, regardless of treatment arm [23,76]. The reasons for this include having a dedicated clinical research team who have more time to care for patients, better monitoring and correction of drug-induced toxicities, and aggressive management of raised intracranial pressure, a common and potentially fatal complication of CM. These quantitative data do not however determine the impact of structural coercion into these trials, nor does it draw on any lived experience of trial participants. This is the focus of our research team who have embedded an ethnographic study into the ongoing AMBIsome Therapy Induction OptimisatioN (AMBITION) study [77].

It is essential to include researchers who are from and/or based in the location where the study is being conducted in a meaningful way. We found that, over time, there was a significant trend of named authors being decreasingly a resident or national of a research site. This finding mirrors the increase in research conducted in LMICs through TRPs and is supported by the significant difference in these 2 domains when comparing trials conducted in HICs and LMICs. This is consistent with broader reviews of global health research in Africa in general which has found that indigenous researchers are frequently “stuck in the middle” [3,15]. For example, Hedt-Gauthier and colleagues found that among general health-related studies published between 2014 and 2016, just 54% of authors were from the country of the paper’s focus and this was 52.9% among the first author. Overall, in this study, we found this to be 70% and 59%, respectively, which is marginally better. There is no doubt that our discipline needs to work much harder to address this inequality, and we acknowledge the authorship of this paper itself lacks the diversity we aspire to. An in-depth discussion is beyond the scope of this article, and we encourage readers to access and actively engage with the growing body of literature on this topic, including discussions on the role of researchers from HICs when it comes to decolonising global health [78–81].

Authorship of CM trial manuscripts was found to have poor representation of female authors. Of all named authors, 29% were female, but a lower proportion were first (26%) and final (13%) author. Although there was no significant change over time, women were better (but still under) represented as authors of trials conducted in LMICs, and as the majority of trials are currently conducted in LMICs, there may be an increase over time. There were however no female authors from LMICs who were listed as the first or final author in the last decade. This finding is consistent with numerous other studies that have found female researchers to be underrepresented in clinical trials and more likely to occupy the middle section of the authorship list [82,83]. This may be partially due to the fact that, in Africa for example, 72% of all physicians are male [84], and in patriarchal societies, female physicians may have competing demands with regard to childcare and family responsibilities [67]. There is an urgent need to ensure female researchers are given the opportunity to gain research experience, be appointed to and supported within senior research roles, and ultimately become eligible for more of the prestigious authorship positions. The Global Health 50/50 initiative provides extensive guidance for individuals and institutions to address gender inequality in the workplace [85].

This is the first systematic review that has described the characteristics of participants in CM interventional trials over time and made a comparison with the general population who develop the disease. There are several limitations to this study. We only included clinical trials in this review, and we acknowledge that clinical trials are not the only useful source of data. There has been a large body of observational work conducted that has informed CM policy which was not included in this review. Observational study participants are more likely to resemble the overall population affected, and, given less stringent requirements in terms of conduct and monitoring, the authors and funders are likely more representative of study locations. There is an urgent need and opportunity with electronic systems to streamline and simplify data collection and monitoring for randomised studies, so that smaller institutions (especially at primary and secondary level) and local investigators are not, in effect, excluded from participation. In addition, there were rare instances where, despite additional efforts, it was not possible to extract data for all variables from published papers. In particular, the earlier studies did not consistently present full screening information. This resulted in some studies being excluded from specific aspects of the analysis which may have led to an inaccurate overall picture.

This work was motivated by the pursuit of equity in global health research. Although broad reviews and commentaries have highlighted concerns regarding representation and inclusion, it is important to acknowledge that each individual disease tends to have its own tight-knit research community and that there may be considerable heterogeneity between groups. We have presented a comprehensive but simple methodology to describe trends in the representation and inclusion of patients and researchers over time. This methodology can be used to help focus our future efforts as we strive for equity. In addition, we now have the foundation of what can be an ongoing monitoring exercise to map our progress over the coming years. We believe that this methodology could be simply adopted and adapted by other research groups.

## Conclusions

HIV-associated CM trials are generally conducted in locations which are heavily affected by the disease. Women and ART-experienced individuals are well represented as participants in clinical trials, but there is an underrepresentation of those with severe CM and those suffering from a relapse. Recent trials have been predominantly conducted in LMICs, and when compared to earlier trials in HICs, there is a tendency for first and senior authors to be nonnational and/or nonresident of the research location. Female researchers are underrepresented in general but particularly as first and senior authors. This paper highlights areas for the CM research community to focus on as we strive for equity.

**Key Learning Points**

- HIV-associated cryptococcal meningitis remains a significant contributor to AIDS-related mortality, and ongoing clinical trials are needed to improve outcomes.
- Trials for cryptococcal meningitis occurred in high-incidence countries, but some highly affected countries have not hosted trials.
- The sex and antiretroviral therapy status of trial participants matched the general population with cryptococcal meningitis, but individuals with reduced consciousness and those suffering a relapse were underrepresented.
- Women were underrepresented as authors, particularly as first and final authors.
- Compared to trials conducted in high-income countries, trials conducted in low- and medium-income countries were less likely to have authors resident in or nationals of the trial location.

**Top Five Papers**

1. Rajasingham R, Smith RM, Park BJ, Jarvis JN, Govender NP, Chiller TM, et al. Global burden of disease of HIV-associated cryptococcal meningitis: an updated analysis. Lancet Infect Dis. 2017;17(8):873–8
2. Azzo C, Tsholo K, Tlhako N, Patel RKK, Leeme T, Jarvis JN, et al. High Mortality in HIV-Associated Cryptococcal Meningitis Patients Treated With Amphotericin B–Based Therapy Under Routine Care Conditions in Africa. Open Forum Infect Dis. 2018;5(11).
3. Heyrana K, Byers HM, Stratton P. Increasing the Participation of Pregnant Women in Clinical Trials. JAMA. 2018;320(20):2077–8.
4. Hedt-Gauthier BL, Jeufack HM, Neufeld NH, Alem A, Sauer S, Odhiambo J, et al. Stuck in the middle: a systematic review of authorship in collaborative health research in Africa, 2014–2016. BMJ Glob Health. 2019;4(5):e001853
5. Global Health 50/50. Power, privilege and priorities: the Global Health 50/50 2020 report. 2020.
