# Correction: Equity in clinical trials for HIV-associated cryptococcal meningitis: A systematic review of global representation and inclusion of patients and researchers

**DOI:** 10.1371/journal.pntd.0011240

**Published:** 2023-03-29

**Authors:** David S. Lawrence, Tshepo Leeme, Mosepele Mosepele, Thomas S. Harrison, Janet Seeley, Joseph N. Jarvis

The Funding statement is incomplete. The complete Funding statement is:

This study was funded as part of the EDCTP2 programme supported by the European Union (grant number TRIA2015-1092), with assistance from the Swedish International Development Cooperation Agency, as well as by funding from the U.K. Department of Health and Social Care, the U.K. Foreign Commonwealth and Development Office, the U.K. Medical Research Council, and Wellcome Trust, through the Joint Global Health Trials scheme (MR/P006922/1).

JNJ is funded by the National Institute for Health Research (NIHR) through a Global Health Research Professorship to JNJ (RP-2017-08-ST2-012) using UK aid from the UK Government to support global health research. The views expressed in this publication are those of the author(s) and not necessarily those of the NIHR or the UK Department of Health and Social Care. The funders had no role in study design, data collection and analysis, decision to publish, or preparation of the manuscript.
